# Complete Genome Sequence of Stenotrophomonas Phage Mendera

**DOI:** 10.1128/MRA.01411-19

**Published:** 2020-01-02

**Authors:** Kameron D. Garza, Heather Newkirk, Russell Moreland, Carlos F. Gonzalez, Mei Liu, Jolene Ramsey, Justin Leavitt

**Affiliations:** a Center for Phage Technology, Texas A&M University, College Station, Texas, USA; Queens College

## Abstract

Stenotrophomonas maltophilia is an emerging opportunistic human pathogen. In this report, we describe the isolation and genomic annotation of the S. maltophilia-infecting bacteriophage Mendera. A myophage of 159,961 base pairs, Mendera is T4-like and related most closely to *Stenotrophomonas* phage IME-SM1.

## ANNOUNCEMENT

Stenotrophomonas maltophilia is a rod-shaped, Gram-negative, nonfermenting, and motile obligate aerobe. Ubiquitous in its distribution, S. maltophilia resides in bodies of water, in sewage, on plants, and in the respiratory tract of humans as an opportunistic pathogen (1). As S. maltophilia is an opportunist, infection caused by the bacterium is a major problem for cystic fibrosis patients (2). In this report, we describe the isolation and genomic annotation of the S. maltophilia-infecting bacteriophage Mendera.

The source for Mendera was filtered (0.2 μm) wastewater collected in Navasota, TX. For isolation, the host S. maltophilia (ATCC 17807) was grown aerobically at 30°C in nutrient broth or agar (BD), and phage was propagated by the soft-agar overlay method (3). Mendera myophage morphology was identified via negative-stain transmission electron microscopy with 2% (wt/vol) uranyl acetate at the Texas A&M Microscopy and Imaging Center (4). Phage genomic DNA was purified with the Promega Wizard DNA clean-up system modified according to the shotgun library preparation protocol (5). Libraries were prepared with the Illumina TruSeq Nano low-throughput kit and sequenced via Illumina MiSeq with paired-end 250-bp reads using V2 500-cycle chemistry. The 222,492 total sequence reads were quality controlled with FastQC (www.bioinformatics.babraham.ac.uk/projects/fastqc), trimmed using the FastX toolkit v0.0.14 (http://hannonlab.cshl.edu/fastx_toolkit/), and assembled into a single raw contig at 11.6-fold coverage using SPAdes v3.5.0 at default parameters (6). Contig accuracy and completeness were confirmed by PCR (forward primer, 5′-GTCACCGCGACTACGATAAG-3′; reverse primer, 5′-GAACGACAGCGAGCATACA-3′) off the contig ends and Sanger sequencing of the product. Possible protein-coding genes were annotated using GLIMMER v3.0 and MetaGeneAnnotator v1.0, and tRNA genes were annotated from ARAGORN v2.36 (7–9). Rho-independent termination sites were annotated using TransTermHP v2.09 (10). To predict protein functions, we used BLAST v2.2.31 sequence similarity searches conducted against the NCBI nonredundant, UniProtKB Swiss-Prot, and TrEMBL databases with a 0.001 maximum expectation value and conserved domain searches via InterProScan v5.33-72 (11–13). ProgressiveMauve v2.4.0 was used to calculate genome-wide DNA sequence similarity (14). PhageTerm was used to predict genomic termini (15). The bioinformatics tools were accessed through the Center for Phage Technology Galaxy and Web Apollo instances hosted at https://cpt.tamu.edu/galaxy-pub/ (16, 17). Unless otherwise stated, all tools were executed using default parameters.

The 159,961-bp double-stranded DNA genome of myophage Mendera has a 97% protein-coding density and a G+C content of 54%, which is lower than the host G+C content of 66.8% (18). In total, 287 protein-coding genes and 23 tRNA genes were predicted. Mendera is related most closely to *Stenotrophomonas* phage IME-SM1, with 198 similar proteins and 92.85% nucleotide identity (GenBank accession no. KR560069). Consistent with its genomic organization and myophage morphology being T4-like, Mendera is predicted to use a headful packaging mechanism.

### Data availability.

The genome sequence and associated data for phage Mendera were deposited under GenBank accession no. MN098328, BioProject no. PRJNA222858, SRA no. SRR8893604, and BioSample no. SAMN11414489.
